# Global Cardiac Surgery—Accessibility to Cardiac Surgery in Developing Countries: Objectives, Challenges, and Solutions

**DOI:** 10.3390/children10111789

**Published:** 2023-11-06

**Authors:** Salvatore Agati, Ermanno Bellanti

**Affiliations:** Mediterranean Pediatric Cardiology Center, Bambino Gesù Children’s Hospital, 98035 Taormina, Italy; salvatore.agati@opbg.net

**Keywords:** global cardiac surgery, global surgery, surgical accessibility

## Abstract

Cardiac surgery is a modern science in the history of medicine. The impact of cardiac disease, in terms of treatment and prognosis, has made this discipline indispensable to global health. In recent decades, the greatest investment has been dispensed to technological and material improvements to increase life expectancy. This surgery must address different epidemiological aspects dictated by the geography and economic–social conditions of the global populations. For this reason, it is progressively important to address the cardiac surgery accessibility disparity. Many scientific papers and international meetings have studied how cardiac surgery can be more accessible in various countries around the world. In this review, we analyze all the challenges, solutions, and suggestions that can make this surgery accessible to the entire global population, with the purpose of reducing its disparity across all seven continents. For a long time, high-income countries have invested in technological capabilities and experimental advancements without caring about unequal access in the rest of the world. We believe that it is time to reverse this growth trajectory, placing the accessibility and distribution of surgical science as a priority, which is significant for the right to health of all people worldwide. This is the real new challenge in cardiosurgery.

## 1. Introduction

Machteld Huber and co. defined health as the ability to be adept and self-manage in the face of social, physical, and emotional challenges [1]. With this definition, health becomes a “collective value” and no longer an individual one, and this outcome is only possible with an equitable distribution of rights and resources. Therefore, health is created when individuals, families, and communities have income, education, and full accessibility to basic rights to control their lives. In addition, their needs and rights are supported by systems, environments, and policies that enable and facilitate better health [2,3].

JM’s latest *Dictionary of Public Health* [4] teaches us that “*Health*” cannot be defined through the achievement of an absolute standard, but rather immersed in a social and community context. In 1990, the WHO defined health inequalities as “*systematic differences in health status between different socio-economic groups; such inequalities are socially produced (hence modifiable) and unjust*”. This statement emphasizes systematicity as opposed to randomness in “social inequality in health, outlining perspectives a solution at the community rather than individual level”. Health disparities, within and between countries, are thus judged to be unjust, avoidable, and unnecessary; they systematically burden populations made vulnerable by the social, political, economic, and legal structures present in these contexts. Brevan & co. [5] proposes the following concept of “*equity in health*”: opportunities for fair and equitable access to services that provide the opportunity to be as healthy as possible. This requires the removal of barriers such as poverty and discrimination. It is for these reasons that health inequalities are defined as systematic, avoidable, and unfair differences that can be observed between individuals, between social groups, or within the same population. Despite the considerable attention given to the problem of health inequalities since the 1980s, significant differences within and between countries still exist today.

This text aims to examine the disparities present in accessibility to cardiac surgery, especially in developing countries. 6 billion people who live in geographic areas without cardiac surgery access is the evidence of disparity [6].

Despite numerous warnings to the political and social world by the WHO, cardiovascular disease remains the leading cause of death worldwide. It is responsible for 17.5 million deaths per year, 80% of which occur in low- and middle-income countries (LIMCs) [7].

Considering that access to cardiac surgery is absent in low-income countries (LICs) and limited in middle-income countries (MICs), it appears that more than 75% of the world’s population has limited/absent access to a health service that, if provided with adequate infrastructure, human resources, and financial coverage, could prevent a huge number of deaths. “Global cardiac surgery” refers to the area of study, research, practice, and advocacy that prioritizes improving health outcomes and achieving health equity worldwide and for all people with heart disease. The proportions of this absurd difference are highlighted by the observation that in sub-Saharan Africa, excluding South Africa, there is one cardiac surgery center for every 38 million people. In contrast, in North America and Europe, there is one for every 120,000 people [8]. To redress this global socio-health imbalance, global cardiac surgery aims to map current global cardiac surgery capacity, support health system strengthening, and establish a framework for developing sustainable programs to reduce the lack of accessibility to cardiac surgery. The success of this health utopia is linked to the development and strengthening of cardiac surgery systems as part of broader health system strengthening through national surgery programs. Only the integration of national health systems and their subsequent strengthening will move cardiac surgery toward universal health coverage. In addition, a collaborative effort between *non-governmental organizations* (NGOs), or other associations, and local governments is needed to facilitate adequate cardiac care in LICs. In developing countries, the requirements for cardiac surgical care to ensure equitable and qualitative responses are manifold: infrastructure, the choice of geographically favorable areas for strengthening hospital presidia, and qualified and trained human resources. The solutions being proposed include specific cardiac learning programs, the identification of non-conflict zones that are designated and secured by local governments, funding to facilitate resource mobilization, and local autonomy. We want to emphasize that disparities are present in all countries, and this is precisely why a comprehensive approach is needed to solve the problems of resource allocation and access to care. This approach must be dictated by shared (government institutions, NGOs, and international programs) and widespread efforts *based on scientific evidence* and guided via data *repositories* and *resources*. Sharing international databases (WHO, local governments, NGOs, and professional societies) is essential to increase research knowledge and innovation, coupled with equitable financial distributions. The prevention and treatment of cardiovascular disease cannot only be a priority for rich countries. Therefore, policies aimed at health system innovation in LICs, coupled with lifestyle changes in high-income countries, should combine to produce an intersectoral benefit to improve global well-being. The 2018 *Cape Town Declaration on Access to Cardiac Surgery in the Developing World* [9] highlights the need, in these countries, for the creation of surgical conditions and nonsurgical elements in local health systems. Pursuing policies that are aimed at strengthening health systems will advance cardiac surgery along with other health services (and not at their expense). The involvement of physicians in social, political, and economic aspects is essential to support the interests of the world’s poorest communities: such outreach must be directed toward promoting the highest quality of health care, regardless of the economic means of the patient being treated.

Only the support of individuals and global cardiac surgery professionals will lead to a substantial reduction in cardiovascular disease mortality worldwide. Such a movement must be global and multidisciplinary, following the simple and well-delineated goals of education, research, and self-sustainability, and resulting in a truly global international coalition that is apt to cooperate and uncluttered by competition. This is the next real challenge of modern cardiac surgery.

## 2. Global Cardiac Surgery

Global cardiac surgery refers to an area of study, research, practice, and advocacy that prioritizes the improvement in health outcomes and the achievement of health equity for all people who are affected by cardiac surgical pathologies in the entire world.

The lack of concern regarding this specific surgery is, unfortunately, the result of the perceived difficulty of cardiac surgery introduction in low-income settings given the complexity of follow-up, the request for intensive human resources training, and high financial costs. Given the growing burden of cardiovascular disease worldwide, including the epidemiological transition from communicable to non-communicable diseases in LMICs, its perception becomes vital for sustainable development and universal health coverage.

Cardiovascular disease is the primary cause of death globally, accounting for approximately 17.5 million deaths each year, 80% of which are in LMICs. Despite the high prevalence of cardiovascular disease in LMICs, the low accessibility to diagnostic services, and the deficiency of health systems, this statistic certainly underestimates the true global state. The data show that around 17 million people die each year from “*predictable surgical conditions*”*,* and only 6% of the 313 million global surgeries take place in LMICs.

After the second half of the 1990s, rheumatic heart disease (RHD) and congenital heart disease (CHD) represented the main challenges in cardiac surgery. During industrialization and after new technology investment, in the Western world, a decrease in rheumatic pathology and an increasingly effective response in congenital surgical pathology were noted. In recent decades, the epidemiological transition has highlighted the shift toward degenerative pathology linked to lifestyle. This change has led to the surgical need for sharp division between high-income countries (HICs) and LMICs. In the first group (HICs), the development of increasingly advanced techniques and progress in interventional modalities constituted an adequate response to the epidemiological change, while the lack of development and training of human resources in LMICs has maintained RHD and CHD at the same levels since the post-war period. This epidemiological panorama is placed within a “*global care capacity deficient*”, associating access to cardio-thoracic surgery proportionally to the economic conditions of the continents. The statistic that best underlines the disparity is that 4.5 million people around the world do not have access to cardiac surgery care. This is the definition of “healthcare accessibility discrimination”.

## 3. Congenital Heart Disease

In the last century, the diagnostic and treatment capabilities of CHD have greatly improved. Several studies show an evident improvement in survival in the more developed regions of the world thanks to early diagnostics and the development of treatment techniques; however, such studies have not had the same success rates in developing regions. For example, advances in pediatric cardiology and heart surgery have made it possible to repair or palliate most CHDs, including complex ones, but many analyses have shown that “*infant survival rates*” depend on the geographic location of the child’s birth.

Screening, prenatal diagnosis, and early treatment tools make it possible to improve the long-term survival of young patients. These advanced treatments are virtually unavailable for more than 90% of babies born in developing countries [2]. In 2016, the General Congress of the United Nations, through the Sustainable Development Goals (SDGs) declaration [3], set the neonatal mortality-freeing goal to less than 12 deaths for every 1000 live births. This objective will be achieved by reducing premature mortality from non-communicable diseases (NCDs) near 30% by 2030. CHD accounts for one-third of all NCDs, and is, therefore, an integral part of reducing preventable childhood deaths. Unfortunately, 90% of the world’s children born with CHD live in places where there is little or no cardiac surgery treatment [4]. This inequality is emphasized by Hoffman and colleagues who show that LMIC infants with severe congenital heart disease and without access to surgical treatment are more likely to die before their fifth birthday than those in HICs [6]. To optimize resources for screening, treatment, and cost-effective treatment strategies, there is a need for an accurate assessment of the absolute and relative burden of congenital heart disease around the world. The *Global Burden of Disease, Injuries, and Risk Factor Study* (*GBD*) is the result of a collaboration of international researchers who annually estimate the prevalence and mortality of CHD all over the world. This report shows that CHD deaths and mortality are highest in LMICs, while rates of improvement have followed rates of economic development, with fatal outcomes decreasing by at least 60% since 1990 in HICs [5]. More in-depth data and studies are needed to update the burden of disease across continents and are essential to monitor progress and encourage funding for sustainable solutions to deliver high-quality care to all children in need. The estimates made in recent years about CHD provide a critical and indispensable point of view concerning the health of the global population. This contributes to a better investment of health resources to increase access to cardiovascular care, especially in less developed countries where progress and resources for care, diagnostics, and rehabilitation have been largely stagnant.

The United Nations Organization has emphasized the priority of reducing premature NCD deaths; however, to achieve this goal, health policies will have to include specific responsibilities aimed at breaking down accessibility barriers by improving access to cardiac diagnosis and treatment. As noted above, most patients with heart disease (CHD or RHD) living in LMICs lack access to quality care. It is well established that the management of CHD and RHD is profitable, justifying the concept of pediatric cardiac care. However, several barrier-filled challenges must be overcome before the average child living in a developing country can access comprehensive heart care. Early diagnosis requires community and healthcare provider awareness of pediatric heart disease, but it is only the beginning. The management of pediatric patients with CHD or RHD requires significant outlay and structure; but, currently, resources are not only limited, but are also sometimes used sub-optimally. The challenges of providing the best care for the average child with heart disease are unparalleled and will lead to innovations and opportunities, but this will require a collective effort by governments, non-governmental organizations, surgeons, and other health professionals for continuous improvement in health care delivery.

The public heart surgery hospital in Benghazi (Libya) is a humanitarian project that our center has been following for three years. This region, inserted in a post-war socio-economic context, refers all patients with CHD to a single private hospital, without the guarantee of equal and widespread accessibility. For this reason, a long-term project has been developed with various regional and international healthcare stakeholders, which promotes cardiac surgery accessibility for cardiac congenital patients [10]. The project’s ambitions aim to achieve regional surgical autonomy, benefitting local staff training, the center’s interconnection, and the targeting the right healthcare investments. This intercontinental network project follows the principles of global cardiac surgery keys, extending international health rights by reducing social and economic disparities in care.

### 3.1. Inequality

The walls to establishing cardiac surgery programs in LICs are numerous and complex, often with the sustainability of investment efforts as the primary challenge.

Recent epidemiological studies have identified non-communicable diseases, in particular cardiovascular disease (CVD), as the first cause of worldwide death.

The greatest burden (up to 80%) of death and frailty from cardiac surgery occurs in LMICs, characterized by a gross national salary per capita of less than USD 10 per year (data from 2004). These alarming tendencies have led to several efforts to develop policies to address this great global contest. Such schemes, by definition, require a combined method that includes preventive and advanced medicine programs that forage into and benefit from each other.

The importance of high-tech medicine, including cardiac surgery, has been highlighted in previous “Institute Medicine reports” as one of the recommendations for promoting cardiovascular health in developing countries. Cardiac surgery is an intensive and costly labor discipline that requires great commitment and planning strategies for structured and funded training. Given the severely limited government budgets for healthcare in LMICs, this objective was initially achieved with the creation of NGOs, including a wide range of specialists. This can be significantly supported through the existing international networks of cardiology, cardiac surgeons, and, above all, cardiac anesthesia and expert societies. In these cases, it is essential to involve the community in both the receiving and donor countries at all stages.

The introduction of disease-specific research programs and population studies significantly increases the strength and impact of such efforts, and, importantly, contribute to the development of local practice guidelines tailored to address problems and specific environmental characteristics.

The ultimate target is to build cardiac centers that are capable of offering high-quality sustainable services that are accessible and are at reasonable prices for entire populations in their catchment areas. The pioneering efforts of Casteñada in Guatemala [11], Carpentier in Vietnam, and Strada in Sudan [12] are inspiring models for offering cardiac surgery to populations in underserved areas. While the load of CD varies significantly among these countries, they share a lack of resources (economic and human), an underdeveloped structure, a lack of precise cost-effectiveness studies that can guide policymakers on the optimal allocation of funds, and, above all, huge unmet clinical demands.

### 3.2. Role of Cardiosurgery Society

In 2018, all the most important cardiac surgery global societies (STS, AATS, EACTS, ASCVTS, and PASCATS) collectively established the Cardiac Surgery Intersociety Alliance (CSIA).

CSIA was created following the guidelines of the first objective of the CTD to be the operational arm of that project. The CSIA is made up of delegates from the major cardio-thoracic societies, the World Heart Federation, and a representative from the pharmaceutical industry. This association is responsible for evaluating, approving, mentioning, and monitoring potential centers in less developed countries to increase access to cardiac surgery; it is also delegated to organize the training of suppliers in these sites.

Thanks to the sharing of opinions related to the problem, the CSIA program has dictated basic principles and criteria in order to implement the dialogue within the selected sites. This is all aimed at respecting transparency, involvement, and sustainability.

When choosing a center, five fundamental aspects are taken into account:

- “Pilot” center;
- Local guarantor;
- Sponsor;
- Training site;
- Educational partner;

The initial strategy is to choose 2–3 starting centers to launch their growth. Once success and feasibility are confirmed, CSIA will increase this plan to other locations. The purpose of this association is to search for sites that are dedicated and determined to establishing long-lasting and sustainable partnerships. The goal of the CSIA is primarily to enable surgeons and cardiac centers in developing countries to increase their long-term clinical and academic capacity through professional and financial support.

However, many other opportunities arise, such as the following:

- *Staff planning*: the distribution of professional human resources across all continental geographical areas is fundamental. The average among adult and pediatric cardiac surgeons is approximately 0.055 per million inhabitants in the poorest countries. In countries with a high GDP, numbers between 7.15 and 9.51 cardiac surgeons/per million inhabitants are seen [13].
  - *There are 179 times more adult cardiac surgeons in HICs than in LICs.*
  - *There are 134 times more pediatric cardiac surgeons in HICs than in LICs.*

These data must be improved and compiled more frequently and updated.

b. *Gender equity*: In HICs, there are relatively few female cardiac surgeons. Among surgical specialties, cardiothoracic is the worst with respect to gender equality. In the USA, only 3% of credentialed cardiothoracic surgeons are female [14]. These inequalities are still more evident within less developed countries, as non-cardiac surgical subspecialties seem to indicate [15].

Women working in cardiac surgery are forced to face much more onerous barriers than their male colleagues, including the wage gap, unconscious bias, educational implementation, development, and domestic arrangement [16]. It is our mandatory responsibility to reduce gender disparity.

c. *Training programs*: the monopolization and cost of training represent, for LICs, educational walls and obstructions.

While HICs have already been proven “frugal” in training cardiac surgeons due to the importance of hands-on education time and a relatively low (compared to other specialties) need for surgeons per million population, LICs are often completely devoid of training programs.

Cardiac surgical training problems are not comparable between HICs and LICs. Many times, in LICs, the only educational possibilities lie in moving to HICs with evident problems in costs, language, and bureaucracy.

### 3.3. Rheumatic Disease

Rheumatic disease is a prototype of inequality in pathologies. Its clinical–statistical relevance is that it is only present in areas of economic–social poverty. The preventive aspect is essential, and a lot of work has been carried out in HICs over the years. For all clinical specifications, please refer [13].

“*Cape Town Declaration on Access to Cardiac Surgery in the Developing World*” has drawn up a program to spread contrast and surgical treatment implementation, together with prevention, in LMICs. All cardiac surgery societies, mainly acting on the distribution of cardiac surgeons and their training, coordinated this effort.

The objective is to “*build local capacity*” through continuous dialogue between the various associations. This dialogue must be inclusive and supported in all its aspects (economic, political, and diplomatic).

The investment in the development of sustainable technologies is a key point to be able to increase distribution both in training and in clinical activity in less developed countries. In a field as complex and expensive as cardiac surgery, the need to optimize results, reduce costs, and increase the volume of procedures (per supplier and center) will be increasingly strong [17].

In this paper, global healthcare inequalities have been described. It is the moral responsibility of every individual involved not to perpetuate this inequality by trying to suppress geographical, ethnic, and economic disparities in cardiac health care accessibility. A person’s health should not be determined by the country or prosperity into which they were born.

## 4. Challenges

The planet’s population is concentrated five times more in LMICs than in HICs.

Although many cardiac surgery programs have developed autonomous centers in LMICs, there is still only coverage for 2% of needs. While this is grossly insufficient for population care in LMICs, it offers a degree of hope for future implementation. These challenges should fuel free public healthcare in LMICs by addressing the existing urban–rural divide. As discussed in the article, many countries are starting processes in this direction, such as Rwanda training its first independent cardiac surgeon [18].

The differentiation in accessibility to a health resource on an economic or geographical basis is no longer tolerable. Every scientific society, biomedical factory, and academy should concentrate its energy and knowledge on reducing this gap via all the means listed in the literature. Cardiac surgery accessibility revolution would boost the credibility of the scientific world by improving global health.

## 5. Conclusions

Cardiac surgery, as a discipline, is destined to remain an area of priority in LMICs.

In HICs, cardiac surgery is now caught between ever-increasing expectations in terms of minimally invasive surgery and the possible disappearance of the discipline in its traditional form (in favor of endovascular procedures). Cardiac surgery in LMICs will be on a growth trajectory for a long time to come.

Caught between the need to reach industrialized countries and the eventual demise of the discipline in its traditional form, cardiac surgery in LMICs will be on a growth trajectory for a long time to come. Although the popularity of cardiac surgery as a surgical specialty has already begun to decrease in HICs, high unmet needs, population growth, and ongoing epidemiological transition will require the continued growth of this lagging discipline in both LICs and MICs.

The challenge for MICs will be not to underestimate the need for rheumatic valve surgery, even if the urban population (consumed by the epidemiological transition), and its command of most of the available resources, obscures the view on an issue that is still significant.

The challenge for LICs will be toward the advancement of their own, sustainable, and independent surgical services, and also their implementation to promote accessibility to patients. The upheavals in the world economy will impact the weakest the most and will make specialist services such as heart surgery “inaccessible and prohibitive”. However, even without these upheavals, the expensive nature of consumables produced at high profit margins in the developed world will maintain a degree of dependence on LICs and MICs that can only be resolved by a collective collaborative effort rather than futile, overblown help. A new level of awareness of the scope and magnitude of this problem has begun to emerge, and the commitment of LICs to establishing independently operated cardiac surgeries, as well as signs of greater awareness in HICs, such as the creation of the CSIA, are promising glimmers of hope.

## Data Availability

The data presented in this study are available on request from the corresponding author. The data are not publicly available due to privacy reasons.
